# Caregivers’ reported views on firearm counseling

**DOI:** 10.1186/s40621-026-00711-3

**Published:** 2026-09-28

**Authors:** Rahul Shah, Sharon Smith, Mohsin Mirza, Sumeet Kadian, Brendan T. Campbell, Kevin Borrup, J. Leslie Knod, Garry Lapidus, Danielle Chenard, Steve Rogers

**Affiliations:** 1 Connecticut Children’s, Hartford, CT USA; 2 University of Connecticut School of Medicine, Farmington, CT USA

**Keywords:** Firearm, Injury prevention, Gun, Safety counseling, Storage, Ammunition

## Abstract

**Background:**

Firearm related injuries are the leading cause of death in children older than one year of age. Safety counseling and prevention are cornerstones of injury prevention. This survey study sought to assess caregivers’ recollection of firearm safety counseling and who provided that counseling in the past two years. It further sought to determine whom they entrusted to provide this information, and their preferred modalities for future counseling. Secondary objectives included assessing the relationship between parental perspectives on who provided this counseling and how this counseling was provided based on presence of a firearm in the home.

**Methods:**

A prospective cross-sectional survey was implemented at a single institution’s pediatric emergency department. Parents of children birth-18 years of age were recruited between November 2020 through May 2022, in a private ED treatment room and the survey was administered using a handheld tablet. Excluded were parents of critically ill/injured children (ESI level 1) or those presenting with a primary psychiatric complaint such as suicidal ideation. Chi-square analyses were performed to assess differences in recollection and perception of firearm counseling measures between caregivers who owned a firearm and those who did not.

**Results:**

A total of 820 of 1,181 of eligible caregivers were recruited (response rate of eligible caregivers: 69.4%). 24.1% of respondents recall receiving counseling on firearm safety within the last two years. 56.1% responded affirmatively that pediatricians ought to counsel parents/guardians on firearm safety. 17.4% reported having a firearm in their home, and these respondents were more likely to have received recent safety counseling. Police departments and pediatricians were the most trusted sources for information; with 58.3% and 55.5% of caregivers identifying them as a highly reputable source of such counseling respectively.

**Conclusions:**

Parents are receptive to firearm related counseling by pediatricians and may benefit from learning about the AAP guidelines for firearm and ammunition storage.

## Background

In 2020 the Center of Disease Control and Prevention (CDC) reported firearm-related injuries as the leading cause of death in children 1–19 years old [1]. Youth suicide has increased at an alarming rate and is now the second leading cause of adolescent death in the United States of America. The presence of a firearm in the home is significantly correlated with an increased risk of suicide death, ranging from two to five times compared to those homes without a firearm [2].

The American Academy of Pediatrics (AAP) offers specific guidelines to general pediatricians regarding the discussion of firearm safety. In its most recent policy statement, the AAP highlighted the importance of “providing anticipatory guidance regarding the increased risk of firearm injuries and deaths with firearms in the home as well as the principles of safer firearm storage.” Citing previous research on the topic, the policy statement also advises caretakers to prevent access of such firearms to children. This may be accomplished by methods such as storing the firearm and ammunition separately, unloaded, and locked [3]. Even a small increase in compliance with these recommendations can have a significant impact on reducing childhood and adolescent firearm-related death [4].

Literature regarding self-reported compliance to firearm safety measures has been mixed with some studies finding a high level of such compliance reaching 90% [5]; while other surveys have found poor compliance [6, 7].

Children and adolescents with mental and behavioral health problems pose an increased risk of suicide. Many children who are experiencing suicidal ideation do not disclose these feelings, and even family and friends may be unaware. In homes with a firearm, when depressive features are identified among children and adolescents, parents are unlikely to reduce access to the gun [8–10].

Reluctance to address an already charged topic may have been further exacerbated by previous legislation seeking to prohibit physicians from discussing firearms with families. While such legislation (since repealed) was isolated to the state of Florida, national implications abound as pediatricians may be confused about their ethical and legal role [11].

This study sought to determine whether parents felt it was appropriate for pediatricians to counsel families on firearm safety. Parents were also asked if they recalled the occurrence of firearm safety counseling performed by their pediatrician, people or institutions they trusted to impart knowledge related to firearm safety, and which modalities (for instance, face-to-face conversations, online videos or modules) they felt would be best to incorporate.

## Methods

This was a prospective cross-sectional study that used a convenience sample and was performed at a single institution’s pediatric emergency department from November 2020 through May 2022. Both English and Spanish-speaking caregivers were recruited. All caregivers were included if their child was younger than 18 years of age, and was assigned an Emergency Survey Index (ESI) level of 2–5. Exclusion criteria included caregivers whose child was in the custody of the Department of Children and Families (DCF), who spent fewer than 20 hours a week providing shelter for the child and those patients who presented to the emergency department with a primary behavioral health concern, as such caregivers are not typically immediately available at bedside. However, it is worth noting that behavioral health patients are screened for access to firearms in our institution. The emergency department was selected as the study site to optimize survey success rate with the rationale that caregivers who are awaiting clinical results or reassessment and have not yet been discharged may be more amenable to complete a voluntary survey.

A total of 29 survey questions were separated into sections; among enrolled participants, survey responses were included in analysis if at least 80% of survey questions from its section were completed. Specific professionals/organizations were considered ‘highly trusted,’ if they were rated 1, 2, or 3 in a list of 14 possible selections.

Subsequent analysis of data demonstrates a variable denominator, which reflects that not all participants answered each question. Patient demographic information was collected as part of the survey.

This study was approved by the Institutional Review Board (IRB, number 00000703). The survey was modified from a previously implemented study [12], and was separated into four sections. The survey was hosted on Qualtrics in both English and Spanish; the Spanish version was created through the help our institution’s Interpretation Services. Chi-square analyses were subsequently completed using *Vassar Stats* to assess differences in recollection and perception of firearm counseling measures between caregivers who owned a firearm and those who did not. Participants were further asked, “Did you know that groups like the American Academy of Pediatrics have developed “best practices” or suggestions on how to safely store firearms/guns in a home?” Multivariable analysis was not performed given the descriptive and exploratory nature of this study. The primary aim was to characterize caregiver perceptions rather than to establish causal relationships or control for confounding. Moreover, data collection did not include all appropriate covariates, such as household income, to allow for a robust multivariable model.

Recruitment was completed by research assistants (not medical staff). Participants were informed that their responses would not be tracked with any identifiers and would not influence their child’s care. Patients and the public were not involved in the design, conduct, reporting, or dissemination plans of our research.

## Results

**Fig. 1:**
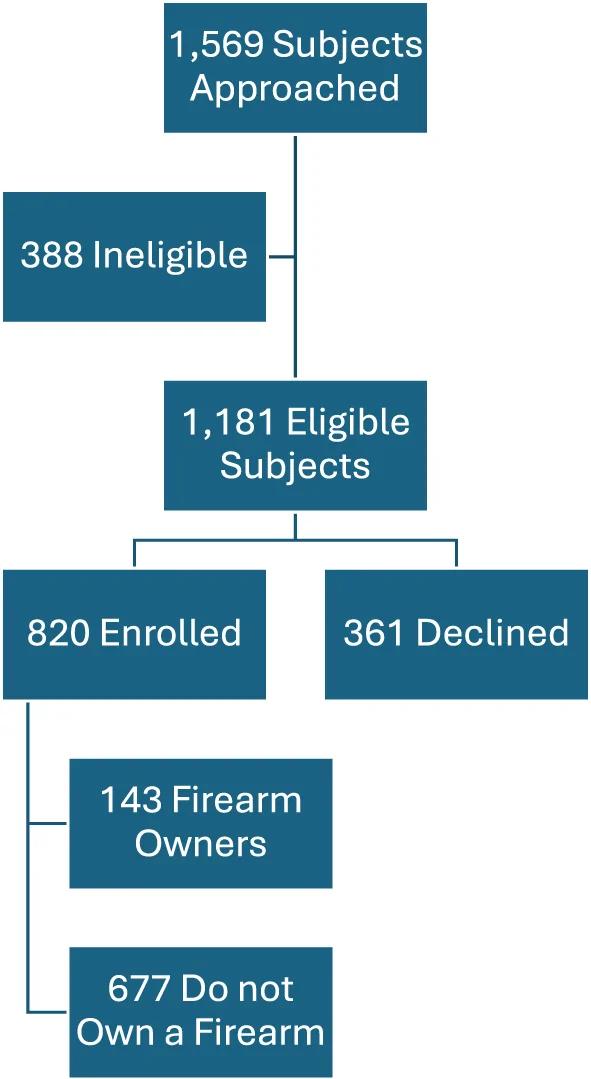
Flow chart that illustrates participant enrollment and those who report having a firearm in their home

A total of 1,569 caregivers were approached, of which 1,181 were determined to be eligible for recruitment. 820 participants were successfully recruited, resulting in a 69.4% response rate of eligible caregivers (Fig. 1).

Of these responses, 751 participants reported being the caregiver who typically took their child(ren) to their pediatric appointments. Of the 765 respondents who answered, 184 (24.1%) recalled having been counseled on firearm safety within the past two years by any person or organization, including their child’s pediatrician. When asked if pediatricians ought to provide information related to firearm safety to parents/guardians, 435 out of 775 respondents (56.1%) responded affirmatively with another 228 (29.4%) reporting they were unsure. A total of 112 respondents (14.5%) reported that pediatricians should not provide safety information, of which 31 (27.7%) were firearm owners.

One hundred forty-three out of 820 participants (17.4%) endorsed having a firearm in their home, with 123 (90.4% of firearm owners) reporting that the firearm was locked and difficult to obtain access. Approximately half of such caregivers (51.9%) caregivers reported that their ammunition was locked separately from their firearm, as the AAP advises and is legally required under Connecticut law (H.R.748, *Ethan’s Law*).

Out of 820 respondents, 235 (28.7%) reported that they have asked a family member or neighbor if there was a firearm in their home. Half (115 of 229 respondents) stated they took some action when they learned the household had a gun. Participants were asked to rank potential trusted sources of firearm safety practices from 1 to 14. Police officers (58.1%), pediatricians (55.5%), their own primary care physician (42.0%) and the NRA (30.5%) were most likely to be picked as among the top three selections, indicating the highest level of trust. Local gun shops (7.0%), websites (1.6%) and social media (0.9%) were the lowest (Table 1).

**Table 1:** Perceptions of firearm counseling and reported firearm safety practices

Perceptions of Firearm Counseling	
Caregiver Who Typically Brings Child to Appointments	751/819 (91.7%)
Recalled Counseling in Prior 2 Years (by pediatrician)	190/774 (24.5%)
Recalled Counseling in Prior 2 Years (any source)	184/765 (24.1%)
Believe Pediatrician Should Counsel	435/775 (56.1%)
Knew AAP Offers Recommendations	245/811 (30.2%)
**Firearm Practices**	
Firearm at Home	143/820 (17.4%)
-*Report Firearm is Locked*	*123/136 (90.4%)*
-*Report Ammunition is Locked* * Separately from Firearm*	*70/135 (51.9%)*
Asked Friends/Family about Firearm Safety Practices in Their Home	235/820 (28.7%)
-*Took Action*	*115/229 (50.2%)*

Nearly half of participants reported that they do not currently receive any information related to firearm safety (347 of 771 respondents − 45.0%). Police departments and the National Rifle Association were the most common sources of information. While nearly half (45.0%) of all respondents denied currently receiving any firearm safety counseling, family members, friends, members of the military, and gun range instructors were each found to provide firearm counseling at a similar rate to pediatricians (Table 2). Participants appeared to favor brochures and face-to-face discussion over online text or videos (Table 3). Overall, most participants rated the police department or their child’s pediatrician as one of the top 3 trusted sources of firearm safety education (Table 4).

**Table 2:** Current sources of firearm safety information

Current Source of Information*	n (% of those who responded) N = 771
No info currently	347 (45.0%)
Police department	132 (17.1%)
NRA	124 (16.1%)
Family members	93 (12.1%)
Gun range instructor	82 (10.6%)
Child’s doctor	81 (10.5%)
Friends	81 (10.5%)
Former military member	77 (10.0%)
Social media	64 (8.3%)
Local gun shop	33 (4.3%)
Other	29 (3.8%)
Other gun association	25 (3.2%)
CT Citizen’s Defense League	24 (3.1%)
My PCP	23 (3.0%)
Websites	21 (2.7%)
No response provided	49

**Table 3:** Preferable methods of dissemination

Preferable Methods of Dissemination	n (% of those who responded) N = 759
Face-to-face discussions	386 (50.9%)
Brochure	316 (41.6%)
Online Modules with Video	153 (20.2%)
Online Modules with Text	102 (13.4%)
None, it should not happen	93 (12.3%)
Other	23 (3.0%)
No response provided	71

**Table 4:** Reported Highly trusted sources of firearm safety information

Ranked As a Top 3 Trusted Source of Information*	n (% of those who responded) N = 770
Police department	449 (58.3%)
Pediatrician	427 (55.5%)
Caregiver’s primary care physician	323 (42.0%)
National Rifle Association	235 (30.5%)
Family members	218 (28.3%)
Former military member	185 (24.0%)
Gun range instructors	151 (19.6%)
Connecticut Citizen’s Defense League	97 (12.6%)
Friends	83 (10.8%)
Other national gun association	58 (7.5%)
Local gun shop	54 (7.0%)
Websites	12 (1.6%)
Other	12 (1.6%)
Social Media	7 (0.9%)
No response provided	50

[* - multiple selections permitted]

Chi square analyses were used to determine potential associations related to having a firearm at home and whether firearm ownership impacted whom caregivers entrusted to provide relevant counseling.

The majority of caretakers did not recall receiving firearm safety information in the last two years, though this figure was slightly improved for caregivers who endorsed possessing a firearm (22.1% vs. 32.6%, *p* < 0.05). A significantly larger portion of firearm owners reported being aware of the AAP recommendations related to firearm safety when compared to those who did not own a firearm (54.6% vs. 25.1%, *p* < 0.05). Our study did not find a significant difference between firearm owners and non-owners in their preference to have pediatricians provide firearm counseling

**Fig. 2:**
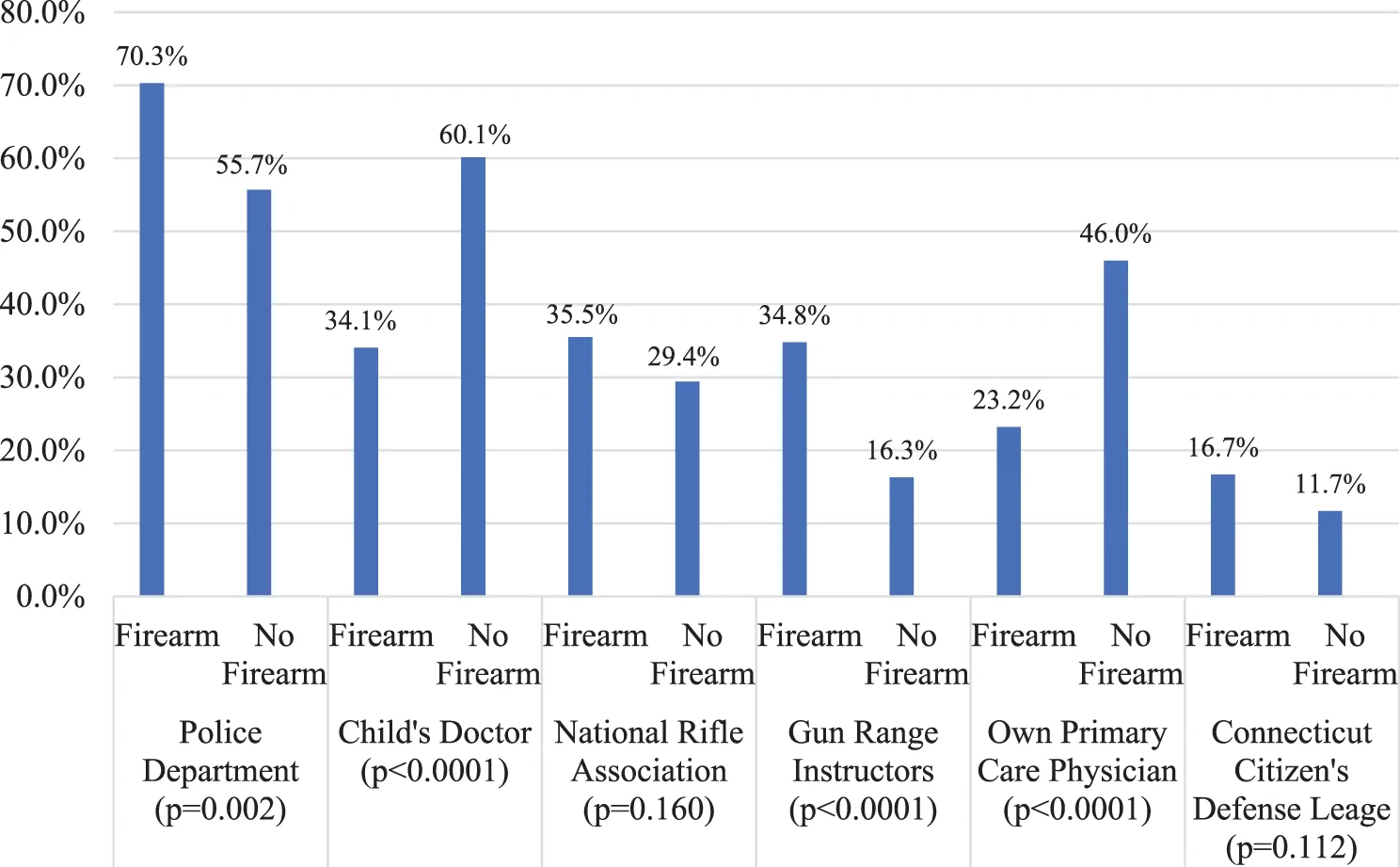
Highly trusted sources of firearm safety information. Percentages reflect proportion of respondents who rated they trusted each professional/organization in the top 3 of 14 potential selections

However, gun owners were less likely to trust their pediatrician to provide relevant information compared to non-owners (34.1% vs 60.1% *p* < 0.05). Conversely, the police department was statistically more trusted as a source of information among firearm owners when compared to those who do not own a firearm (70.3% vs. 55.7%, *p* < 0.05). Firearm owners and non-owners were similar in entrusting the National Rifle Association as a primary source of trusted information (35.5% vs 29.4%, *p* = NS) (Fig. 2, Tables 5 and 6).

**Table 5:** Chi square analyses of caregivers’ awareness and perceptions

	Firearm	No Firearm	p Value
Recalled Receiving Firearm Safety Information within Past 2 Years	44/135 (32.6%)	139/630 (22.1%)	0.009
Awareness of AAP Recommendations	77/141 (54.6%)	168/670 (25.1%)	<0.001
Disclosed Mental Health Concerns for Child	29/162 (17.9%)	128/616 (20.8%)	0.417
Belief that Pediatricians Should Discuss Firearm Safety	79/139 (56.8%)	356/636 (56.0%)	0.863
Reported Highly Trusted Source of Information			
Police Department	97/138 (70.3%)	352/632 (55.7%)	0.002
Child’s Doctor	47/138 (34.1%)	380/632 (60.1%)	<0.001
Own Primary Care Physician	32/138 (23.2%)	291/632 (46.0%)	<0.001
Local Gun Shop	18/138 (13.0%)	36/632 (5.7%)	0.002
Gun Range Instructors	48/138 (34.8%)	103/632 (16.3%)	<0.001
National Rifle Association	49/138 (35.5%)	186/632 (29.4%)	0.16
Connecticut Citizen’s Defense League	23/138 (16.7%)	74/632 (11.7%)	0.112

**Table 6:** Chi square analyses between insurance and firearm counseling and ownership

		p Value
	**Pediatrician Discussed Firearm Safety**	0.003
Public insurance	60/195 (23.5%)	
Private insurance	113/210 (35.0%)	
	**Own firearm**	<0.001
Public insurance	24/283 (7.8%)	
Private insurance	103/297 (25.8%)	

## Discussion

Pediatricians can play a pivotal role promoting childhood safety during their encounters with parents; however, discussion on firearm safety was not recalled by three of four parents in this study. While many parents do not recall having received counseling, it is possible that counseling had occurred. Previous studies offer potential explanations as to why this critical dialogue does not occur; unfamiliarity with best practices and time constraints appear to play significant roles [13]. Over one-third of pediatric trainees reported feeling ill-equipped to discuss firearm safety with families [14, 15]. Conventional forms of firearm safety counseling, such as direct questioning and financial incentives to procure locking devices, may be ineffectual, adding skepticism on the utility of initiating dialogue [16]. Previous research has indicated that adult males are typically the individuals in homes with the most perceived influence over firearm storage practices. When firearm safety is discussed, much of such information is disseminated to a female in the child’s home [17–19].

Prior research found that firearm owners are less likely than non-firearm owners to believe it is appropriate for physicians to offer counseling on relevant best gun safety practices [20]. Our study contradicts this finding; there was no statistical difference between the perceptions regarding pediatrician counseling of those who possess firearms in their home and those who do not.

Caregivers in this study who endorsed having a firearm in their home reported overall mixed compliance with the American Academy of Pediatrics’ firearm safety guidelines. This is in keeping with prior research in Connecticut, with a sizable proportion of those reporting that their ammunition was not locked separately from their firearm [21]. In Connecticut, safe storage laws require firearms and ammunition be locked and stored separately. The majority kept their firearm unloaded and in a locked container or device, nearly half endorsed keeping ammunition in the same device. While this study did not seek to determine family’s understanding of current legislation, it did provide this information to participants who may be storing their firearm and/or ammunition out of such compliance.

Recent research has found that most firearm owners are receptive to being counseled on firearm safety by their pediatrician [22]. This study revealed that just over half of respondents believed that pediatricians should counsel families on firearm safety, however almost half did not endorse their pediatrician provided counseling on gun safety. This may speak to the perceived polarizing nature of this issue. While multiple medical organizations, including the AAP [23] have issued statements indicating that they consider firearm safety a public health issue, it is possible that some people may not share this view. While one reasonable reaction may be to implement more passive forms of counseling, such as through the use of online modules, rather than face-to-face dialogue. Our research suggests that in-person may be preferred. However, if clinical time restraints are the pediatricians’ noted limitation for providing firearm safety counseling, as needed for a face-to-face dialogue, the second preferred method to receive such education was a brochure. Considering gun violence as a public health issue could depoliticize the issue. Further study on the general public’s current perception of gun violence as a health issue could provide further insight on how to bridge this potential divide.

Parents who have a firearm at home had similar perceptions to pediatrician counseling on gun safety when compared to parents who do not. This may speak to the benefit of universal counseling, though it is important to recognize that those with firearms overall trusted pediatricians less to provide high-quality firearm safety information.

Caregivers with a firearm in their home reported a higher level of trust in police officers providing firearm safety counseling compared to those who did not. Police were the overall most trusted group in this study, suggesting there may be opportunities for pediatricians and other injury prevention officials to partner with police. Police may serve as useful educators to both the general public and pediatricians who seek to learn more about various safety devices and their use. This may empower pediatricians to discuss gun safety more confidently with parents. While our study did not assess why police were the most entrusted, we posit this is due to their first-hand training and use of firearms. This lived experience may not be shared by all pediatricians and may be difficult to replicate.

The results of this study have several limitations. First, the study was conducted in a single site in Connecticut. Firearm ownership and practices may vary significantly across regions, and our results may not be generalizable. Recall bias is another possibility; caregivers may not be able to accurately indicate if they have been screened in the past. Caregivers’ ability to accurately recall such screening may be further compromised by the inherent stress of presenting to the emergency department with a sick child. Response bias may occur if certain subpopulations are uncomfortable being asked about firearms in this survey.

Directions for future study include determining whether firearm owners in urban settings are more likely to keep a firearm for personal or property protection when compared to those who live in a more rural setting. In addition, determining how young adolescents themselves perceive firearm education will be a useful future avenue of study [24, 25].

## Conclusions

Based on this study, just over half of caregivers in our convenience sample are following AAP firearm recommendations regarding safe storage of guns and ammunition. Overall, we found that caretakers who own a firearm are more likely to recall counseling on safety measures, equally as likely to believe their pediatrician should offer such counseling, and less likely to entrust their pediatrician to provide them with high-quality information when compared to caretakers who do not own a firearm. Pediatricians and police were viewed as trusted sources of information on firearm safety.

## Data Availability

The datasets used and/or analyzed during the current study are available from the corresponding author on reasonable request.

## Abbreviations

AAP: American Academy of Pediatrics
DCF: Department of Children and Families (Connecticut)
ESI: Emergency Survey Index
IRB: Institutional Review Board

## Declarations

### About this supplement

This article has been published as part of Injury Epidemiology Volume 13 Supplement 1, 2026: Proceedings of the 30th Annual Injury Free Coalition for Kids® Conference: Forging New Frontiers: injury Prevention in Evolving Environments. The full contents of the supplement are available online at https://link.springer.com/journal/40621/volumes-and-issues/13-1/supplement.

## References

[1] Goldstick JE, Cunningham RM, Carter PM. Current causes of death in children and adolescents in the United States. N Engl J Med. 2022;386(20):1955–56. 10.1056/NEJMc2201761. PMC10042524 35443104

[2] Wolk CB, Jager-Hyman S, Marcus SC, Ahmedani BK, Zeber JE, Fein JA, et al. Developing implementation strategies for firearm safety promotion in paediatric primary care for suicide prevention in two large US health systems: a study protocol for a mixed-methods implementation study. BMJ Open. 2017;7(6):e014407. 10.1136/bmjopen-2016-014407. PMC5541509 28647722

[3] Lee LK, Fleegler EW, Goyal MK, Doh KF, Laraque-Arena D, Hoffman BD. Firearm-related injuries and deaths in children and youth: injury prevention and harm reduction. Pediatrics. 2022;150(6). 10.1542/peds.2022-060070. 36207776

[4] Grossman DC, Mueller BA, Riedy C, Dowd MD, Villaveces A, Prodzinski J, et al. Gun storage practices and risk of youth suicide and unintentional firearm injuries. JAMA. 2005;293(6):707–14. 10.1001/jama.293.6.707 15701912

[5] Monuteaux MC, Azrael D, Miller M. Association of increased safe household firearm storage with firearm suicide and unintentional death among US youths. JAMA Pediatr. 2019;173(7):657. 10.1001/jamapediatrics.2019.1078. PMC6515586 31081861

[6] Fuzzell LN, LaJoie AS, Smith KT, Philpott SE, Jones KM, Politi MC. Parents’ adherence to pediatric health and safety guidelines: importance of patient-provider relationships. Patient Educ Couns. 2018;101(9):1570–76. 10.1016/j.pec.2018.05.003. PMC6057837 29731179

[7] Berrigan J, Azrael D, Hemenway D, Miller M. Firearms training and storage practices among US gun owners: a nationally representative study. Inj Prev. 2019;25(Suppl 1):i31–38. 10.1136/injuryprev-2018-043126. 30878975

[8] Hamilton D, Lemeshow S, Saleska JL, Brewer B, Strobino K. Who owns guns and how do they keep them? The influence of household characteristics on firearms ownership and storage practices in the United States. Prev Med. 2018;116:134–42. 10.1016/j.ypmed.2018.07.013. 30081133

[9] Scott J, Azrael D, Miller M. Firearm storage in homes with children with self-harm risk factors. Pediatrics. 2018;141(3). 10.1542/peds.2017-2600. 29467279

[10] Simonetti JA, Mackelprang JL, Rowhani-Rahbar A, Zatzick D, Rivara FP. Psychiatric comorbidity, suicidality, and in-home firearm access among a nationally representative sample of adolescents. JAMA Psychiatry. 2015;72(2):152–59. 10.1001/jamapsychiatry.2014.1760. 25548879

[11] Simonetti JA, Theis MK, Rowhani-Rahbar A, Ludman EJ, Grossman DC. Firearm storage practices in households of adolescents with and without mental illness. J Adolesc Health. 2017;61(5):583–90. 10.1016/j.jadohealth.2017.05.017. 28822681

[12] Kuehn BM. Battle over Florida legislation casts a chill over gun inquiries. JAMA. 2015;313(19):1893–95. 10.1001/jama.2015.3410. 25901487

[13] Campbell BT, Thaker S, Fallat ME, Foley DS, McClure E, Sakran JV, et al. A multicenter evaluation of a firearm safety intervention in the pediatric outpatient setting. J Pediatr Surg. 2020;55(1):140–45. 10.1016/j.jpedsurg.2019.09.044 31753607

[14] Olson LM, Christoffel KK, O’Connor KG. Pediatricians’ involvement in gun injury prevention. Inj Prev. 2007;13(2):99–104. 10.1136/ip.2006.012401. PMC4131854 17446249

[15] Solomon BS, Duggan AK, Webster D, Serwint JR. Pediatric residents’ attitudes and behaviors related to counseling adolescents and their parents about firearm safety. Arch Pediatr Adolesc Med. 2002;156(8):769–75. 10.1001/archpedi.156.8.769. 12144366

[16] Kwong JZ, Gray JM, Rein L, Liu Y, Melzer-Lange MD. An educational intervention for medical students to improve self-efficacy in firearm injury prevention counseling. Inj Epidemiol. 2019;6(Suppl 1):27. 10.1186/s40621-019-0201-3 PMC6616463 31333993

[17] Grossman DC, Cummings P, Koepsell TD, Marshall J, D’Ambrosio L, Thompson RS, et al. Firearm safety counseling in primary care pediatrics: a randomized, controlled trial. Pediatrics. 2000;106(1 Pt 1):22–26. 10.1542/peds.106.1.22 10878144

[18] Azrael D, Miller M, Hemenway D. Are household firearms stored safely? It depends on whom you ask. Pediatrics. 2000;106(3):E31. 10.1542/peds.106.3.e31. 10969115

[19] Coyne-Beasley T, Johnson RM. Gun storage-Who’s the right target? Pediatrics. 2001;108(3):823. 10.1542/peds.108.3.823-a. 11548766

[20] Payton E, Khubchandani J, Thompson A, Price JH. Parents’ expectations of high schools in firearm violence prevention. J Community Health. 2017;42(6):1118–26. 10.1007/s10900-017-0360-5. 28527100

[21] Betz ME, Azrael D, Barber C, Miller M. Public opinion regarding whether speaking with patients about firearms is appropriate: results of a National survey. Ann Intern Med. 2016;165(8):543–50. 10.7326/M16-0739. 27455516

[22] Schenck C, Wilson M, Tiyyagura G, Bechtel K. Parental attitudes, beliefs, and practices related to firearm storage: a qualitative study. Inj Epidemiol. 2022;9(Suppl 1):35. 10.1186/s40621-022-00400-x PMC9768769 36544237

[23] Kandeepan A, Lee J, Bagdure D, Garber N, Day J, Holloway A, et al. Firearm screening in pediatric patients. Front Pediatr. 2024;12:1415612. 10.3389/fped.2024.1415612 PMC11228932 38978836

[24] Monroe KK, Fried SQ, Rubin A, Markman LR, Shefler A, McCaffery H, et al. Firearms screening in the pediatric inpatient setting. Hosp Pediatr. 2020;10(1):37–42. 10.1542/hpeds.2019-0040 31792099

[25] Schwebel DC, Long DL, Gowey M, Severson J, He Y, Trullinger K. Study protocol: developing and evaluating an interactive web platform to teach children hunting, shooting and firearms safety: a randomized controlled trial. BMC Public Health. 2021;21(1):308. 10.1186/s12889-021-10345-3. PMC7866873 33549072

